# Carnosine Alleviates Knee Osteoarthritis and Promotes Synoviocyte Protection via Activating the Nrf2/HO-1 Signaling Pathway: An In-Vivo and In-Vitro Study

**DOI:** 10.3390/antiox11061209

**Published:** 2022-06-20

**Authors:** Prabhakar Busa, Sing-Ong Lee, Niancih Huang, Yaswanth Kuthati, Chih-Shung Wong

**Affiliations:** 1 Department of Anesthesiology, Cathay General Hospital, Taipei City 106, Taiwan; prabhakar.busa01@gmail.com (P.B.); onguitar79@gmail.com (S.-O.L.); yaswanthk1987@gmail.com (Y.K.); 2 Department of Anesthesiology, Tri-Service General Hospital, Taipei City 114, Taiwan; niancih@hotmail.com; 3 National Defense Medical Center, Graduate Institute of Medical Sciences, Taipei City 114, Taiwan

**Keywords:** knee osteoarthritis, carnosine, interleukin-1β, reactive oxygen species, nuclear factor erythroid 2-related factor, heme oxygenase, matrix metalloproteinases proteins, fibroblast-like synoviocytes

## Abstract

The most common joint disease in the elderly is knee osteoarthritis (OA). It is distinguished by cartilage degradation, subchondral bone loss, and a decrease in joint space. We studied the effects of carnosine (CA) on knee OA in male Wistar rats. OA is induced by anterior cruciate ligament transection combined with medial meniscectomy (ACLT+MMx) method and in vitro studies are conducted in fibroblast-like synoviocyte cells (FLS). The pain was assessed using weight-bearing and paw-withdrawal tests. CA supplementation significantly reduced pain. The enzyme-linked immunosorbent assay (ELISA) method was used to detect inflammatory proteins in the blood and intra-articular synovial fluid (IASF), and CA reduced the levels of inflammatory proteins. Histopathological studies were performed on knee-tissue samples using toluidine blue and hematoxylin and eosin (H and E) assays. CA treatment improved synovial protection and decreased cartilage degradation while decreasing zonal depth lesions. Furthermore, Western blotting studies revealed that the CA-treated group activated nuclear factor erythroid 2-related factor (Nrf2) and heme oxygenase (HO-1) and reduced the expression of cyclooxygenase-2 (COX-2). FLS cells were isolated from the knee joints and treated with IL-1β to stimulate the inflammatory response and increase reactive oxygen species (ROS). The matrix metalloproteinase protein (MMP’s) levels (MMP-3, and MMP-13) were determined using the reverse transcription-polymerase chain reaction (RT-PCR), and CA treatment reduced the MMP’s expression levels. When tested using the 2′,7′-dicholorodihydrofluroscene diacetate (DCFDA) assay and the 5,5′,6,6′-tetracholoro-1,1′,3,3′-tertraethylbenzimidazolcarboc janine iodide (JC-1) assay in augmented ROS FLS cells, CA reduced the ROS levels and improved the mitochondrial membrane permeability. This study’s investigation suggests that CA significantly alleviates knee OA both in vitro and in vivo.

## 1. Introduction

Knee osteoarthritis (OA) is a chronic degenerative disease that is the foremost cause of pain and disability in elderly people [1]. Inflammation of multiple tissues, such as the synovial membrane, articular cartilage, and subchondral bone, is the principal pathological effect of OA [2]. Even though the precise mechanism of OA pathogenesis remains unknown, many factors contribute to the onset and progression of OA, along with mitochondrial damage, oxidative stress, changes in the chondrocyte’s inflammatory activity, and abnormal extracellular matrix catabolism [3]. Articular cartilage (AC) is mainly composed of vascular, lymphatic, and neural tissue, as well as chondrocytes [4]. The extracellular matrix (ECM), which is composed of 70% water and organic components, structures AC. The AC acts as an excellent lubricant for bone surfaces. AC absorbs movement-induced stress and provides a smooth platform for efficient joint motions. AC is composed of type II collagen and aggrecans proteoglycans, which work together to increase bone strength and flexibility [5,6].

Inflammatory mediators have long been known to play an essential role in cartilage ECM degradation [7]. OA patients have augmented IL-1β, IL-6, and TNF-α levels. IL-1β is an important mediator of joint inflammation, and enhanced levels in the chondrocytes and synoviocytes can be detected in the early stages of OA [8]. Increased levels of inflammatory mediators cause an abnormal chondrocyte phenotype, which directly restricts ECM collagen and aggrecan protein synthesis [9]. Inflammation causes cartilage component degradation by enhancing the release of MMP’s (MMP-3 and MMP-13) and aggracanase enzymes [10]. Recent studies have shown that IL-1β is capable of inducing its secretion as well as stimulating the synthesis of other inflammatory mediators [11]. When IL-1β and TNF-α bind to their receptors, an inflammatory signaling cascade is activated, which enhances inflammation and catabolism by increasing the expression of adhesion molecules [12]. The increased synthesis of cytokines, as well as increased expression of MMP’s family enzymes, can result in ECM degradation [13]. IL-1β associates with Th17 and NK22 cells for neutrophils recruitment into the tissue, which helps to promote OA [14,15]. Furthermore, IL-1β secretion influences and enhances the generation of catabolic and inflammatory factors, such as metalloproteinase with thrombospondin 5 (ADAMTS5), MMP-3, 13, COX-2, NO, inducible nitric oxide synthase (iNOS), TNF-α, interleukin-6 (IL-6), and prostaglandin-E2 (PGE_2_). These factors could be potential treatment targets for OA. As a result, these discoveries are being used to develop an effective treatment strategy for OA caused by IL-1β-induced inflammation [16,17,18].

Recent studies have shown that the nuclear factor kappa B (NF-κB) pathway regulates inflammatory and catabolic genes in chondrocytes via IL-1β [19]. In response to IL-1β activation, the NF-κB inhibitor pathway is phosphorylated and degraded. Subsequently, the release of p65 dimers promotes p65 translocation into the nucleus and binding to specific genes, causing inflammation [20]. Furthermore, activation of the Nrf2/HO-1 signaling transduction pathway inhibits the pro-inflammatory effects of NF-κB by increasing the expression of antioxidant regulator proteins, such as Nrf2, NAD (P)H-quinone oxidoreductase-1, and HO-1 [21]. A recent report demonstrated that an increase in Nrf2 levels can prevent OA progression in rats by inhibiting the NF-κB pathway [22]. In another study, a treatment strategy that focused on the Nrf2/HO-1 pathway reduced inflammation and cartilage degradation in OA patients, providing a breakthrough for OA treatment [23,24].

The synovium lines the joint cavity, and its essential function is to generate synovial fluid, which allows the joint to move freely. The concentrations of synovial fluid components, such as lubricin and hyaluronic acid, decrease with the progression of OA, influencing the cartilage integrity. The synovium is composed of two main regions: the synovium lining and the synovium sublining layers. Macrophages and fibroblast-like cells are composed of the synovial lining. The synovial sublining is composed of fibroblasts, macrophages, adipose tissue cells, and blood vessels, with fewer lymphocytes [25]. In the early and late stages of OA, synovial inflammation has been observed. Following that, cartilage degradation and bone erosion occur over time because of MMP’s and cytokine release (IL-1β and TNF-α). A role for macrophages and their linked inflammatory cytokines in knee OA has also been identified [26,27,28].

Despite this, many therapeutic agents are available for treating knee OA, such as non-steroidal anti-inflammatory and antioxidant agents [29]. Because these drugs have side effects, there is an urgent need to develop novel therapeutic agents for OA treatment [30]. Methionine and arginine, for example, are amino acids that play a crucial role in the treatment of inflammation and knee OA [31,32]. CA is a dipeptide that contains β-alanine and L-histidine. CA’s anti-inflammatory capacity in autoimmune disease has been investigated [33]. CA’s antioxidant, toxic metal ion chelating nature, and anti-glycating properties have all been well documented [34]. A recent study on the effect of CA on oxidative stress in HK2 cells (human kidney tubular epithelial cells) found that CA may reduce NADPH oxidase (NOX-4) expression while increasing total superoxide dismutase (T-SOD) levels [35]. Thus, it significantly reduces the ROS intracellularly and oxidative stress of cells [36]. It was discovered that it directly reacts with the superoxide anions as ascorbic acid under ROS physiological conditions. It was discovered to reduce oxidative damage and enhance the antioxidant capacity of various antioxidant enzymes, such as superoxide dismutase (SOD), catalase (CAT), reduced glutathione levels (GSH), and glutathione peroxidase (GPx) [37,38]. Recent research has shown that CA can be used as a supplement to reduce cytokine release and, as a result, inflammation in humans [39].

Furthermore, CA can also reduce renal IL-6 and TNF-α in rats exposed to nickel-induced nephrotoxicity [40]. Higher levels of pro-inflammatory cytokines (IL-6 and TNF-α) are usually related to insulin resistance and changes in insulin sensitivity in diabetic patients [41]. These cytokines can also be used as markers of renal failure in the context of diabetic neuropathy. CA regulates the above effects by inhibiting pro-inflammatory cytokine production and reducing inflammation [42]. CA and histidine supplementation via drinking water and diet protected diabetic Balb/cA mice by significantly suppressing pro-inflammatory cytokines, such as IL-6 and TNF-α, and inhibiting diabetes-induced neuropathy [43]. CA has recently been shown to suppress and slow aging in cultured human fibroblasts [44]. Tarnopolsky et al. invested in the carnosine and taurine potential in the skeletal muscle fibers of elderly OA patients. CA may have reduced type II fibers and intracellular buffering capacity by inhibiting the glycogen phosphatase and type I or type II myosin ATPase activities. This fluctuating CA content reduced physical activity and hastened the progression of OA [45]. Nonetheless, the anti-inflammatory activity of CA in OA has been narrowly studied [46].

**Figure 1:**
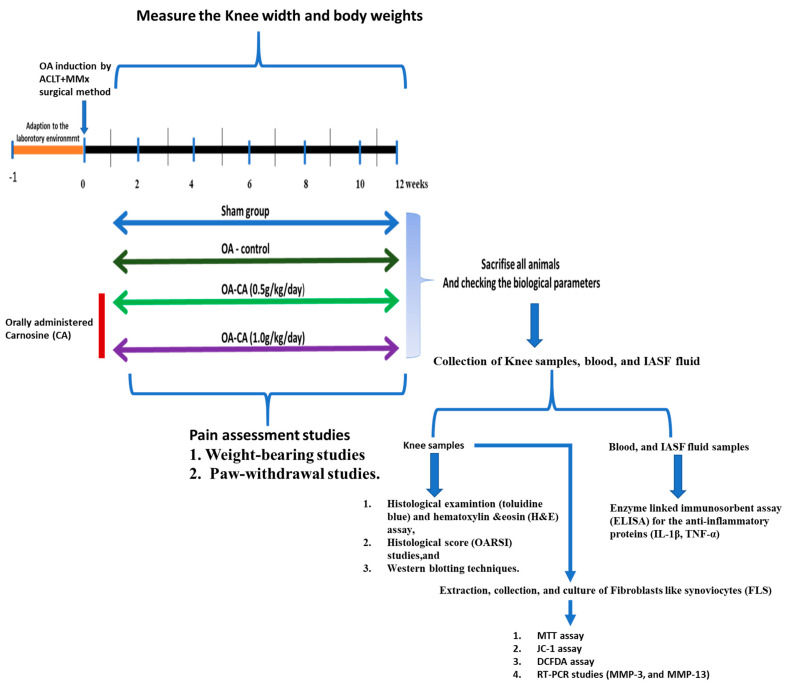
Photographic representation of the experimental design, treatment plan, and collection of knees, blood, and IASF samples for inspection of different parameters.

In this study, we have investigated the role of CA in overcoming knee OA via in vivo and in vitro studies, as shown in Figure 1. Knee OA was induced using the ACLT + MMx method, followed by CA supplement for 12 weeks. Pain-related behavioral studies (weight-bearing and withdrawal studies), knee-width change measurements, and body weight changes were measured for 12 weeks. Furthermore, toluidine blue assay and H and E staining assays were performed to examine the histopathological changes in knee OA tissue. The levels of pro-inflammatory cytokines (IL-1β and TNF-α) in the serum and IASF fluids were then determined using ELISA. Furthermore, Western blotting was performed in the extracted knee samples to check the activation of the Nrf2/HO-1 signaling and COX-2 protein expression. FLS cells extracted from the knee-joint tissues were used in the in vitro studies. Cell viability studies were performed in the presence and absence of the ROS stimulant IL-1β. Next, RT-PCR was utilized to determine the levels of MMP-3 and MMP-13 in the ROS-stimulated FLS cells. CA’s antioxidant capacity was tested using JC-1 and DCFDA assays.

## 2. Materials and Methods

### 2.1. Experimental Animals

Throughout the experiments, adult male Wistar rats (8 weeks old) were kept in pathogen-free conditions. BioLASCO Taiwan Co. Ltd. Provided the rats (Yilan, Taiwan). During the experiments, the rats were housed at Cathay Medical Research Institute (CMRI) and fed a standard laboratory diet (LabDiet; PMI Nutritional International, St. Louis, MO, USA) and dd-water ad libitum. The animal room environment was maintained at room temperature (22 ± 2 °C) and humidity levels of 55–60% with a 12 h light/12 h dark cycle. After a week of acclimation to the animal house environment, the experimental knee OA study will begin. All animal experiments were inspected by Cathay General Hospital’s Institutional Animal Care and Use Committee (IACUC). Animal studies were conducted in accordance with the IACUC ethics committee’s guidelines (IACUC number: CGH-IACUC-111-002).

### 2.2. ACLT + MMx Method Was Used to Treat Rat-Induced Knee OA

**Figure 2:**
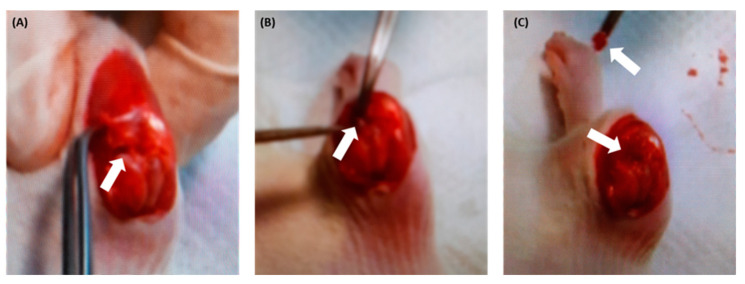
Photographic representation of knee OA induced by ACLT + MMx surgery. Male Wistar rats were used for surgery (**A**) exposure of the knee and showing ACLT and MMx before the ACLTtranssection and MMx removal, (**B**) ACLT transection, and (**C**) MMx removal method to destroy the integrity of the knee-joint structure.

Following the previous reports, OA was induced in rats [47,48,49]. The ACLT + MMx method was employed to induce OA in rats, and surgery on the right knee joint was performed (Figure 2). Rats were anesthetized with 5% isoflurane (Panion and BF Biotech Inc, Taipei, Taiwan) and placed in an induction chamber with 2% isoflurane maintained with a custom-made facemask. The animals were properly anesthetized before being placed on the surgical platform, where the right knee joint was shaved, and the surgery was performed. In brief, an incision was made on the medial aspect of the joint capsule, the anterior cruciate ligament (ACLT) was transected, and the medial meniscus (MMx) was removed. The surgically induced OA joints were rinsed with normal saline solution before the joint capsule was sutured closed and sterilized with iodine solution. Cefazolin (Sigma-Aldrich, Taiwan; Merck and Co. Neihu, Taipei, Taiwan) (100 mg/kg/day) was administered intramuscularly (IM) for 3 days to maintain a hygienic condition. Sham rats had an incision in the medial aspect of the joint capsule made to expose the anterior cruciate ligament (ACL), but the ACL was not transected, and the MMx was not removed.

### 2.3. CA Administration

The animals were given CA (Sigma-Aldrich, Darmstadt, Germany) (0.5 and 1.0 g/kg/day) orally for 12 weeks after knee OA was induced. The knee widths were measured using a caliper (AA847R, Aesculap, Eindhoven, Netherlands). For 12 weeks, incapacitance tests were used to evaluate the weight-bearing pain studies. Finally, animals were sacrificed, and their knees were embedded in a 4% paraformaldehyde solution and used in subsequent experiments.

### 2.4. Measurements of Knee Width and Weight-Bearing Tests

A caliper (AA847R, Aesculap, Eindhoven, and The Netherlands) was used to measure the width of the knee, and the width of the non-surgery knee served as the baseline. The data were expressed as the ∆ knee width (nm). The values from the surgery knee mean value of knee width minus the non-surgery knee width mean value and subtracted by the surgery rat’s mean volume of knee width.

A weight-bearing test was also performed to check for postural changes using an incapacitance apparatus (Linton Instrumentation, Norfolk, UK). The rats were placed on their hind paws in a box with an inclined plane (65° from the horizontal), and the total glass apparatus was kept on the incapacitance apparatus. The data is expressed as the weight difference between the non-surgical limb and the surgical limb (∆ Force, g) [50].

### 2.5. Paw-Withdrawal Examination

A Dynamic Plantar Aesthesiometer was used to assess paw sensitivity (Ugo Basile, Comerio, Italy). The animals were housed in a polycarbonate chamber with a metal mesh floor. Animals were acclimatized for 20 min. The withdrawal threshold of the OA or sham rats was determined by gradually lowering the mmHg from 1 to 50 g via a metal filament (0.5 mm) aimed at the plantar region. The paw reflexes were measured in triplicates every 2 min, and the average was set at 50 g as the cut-off threshold to prevent paw injury [51].

### 2.6. Histology Studies

The knee joints were extracted and immersed in a 4% paraformaldehyde solution (Sigma-Aldrich, Taiwan; Merck and Co., Neihu, Taipei, Taiwan). The knees were then decalcified for 4 weeks in 12.5% EDTA (pH-7.0) (Sigma-Aldrich, Darmstadt, Germany). Next, the joints were embedded in paraffin blocks and cut into 5 μm thick (200 μm) sections for tissue pathological examination. Finally, morphological changes were assessed using Toluidine blue/fast green and H and E staining assays. The stained sections were photographed using the ZEISS Axioscan Z1 imaging system (Jena, Germany). The osteoarthritis research society international (OARSI) scoring method was used to assess the severity of articular damage. We checked the cartilage matrix loss width, tibia cartilage degradation, zonal depth ratio, significant cartilage degeneration width, and synovial degradation in histological sections [52].

### 2.7. Measurements of Cytokines

Before scarifying the animals, blood samples were collected via the tail vein method.

The blood was centrifuged at 3000× *g* for 15 min to collect the serum samples, which were then stored at −80 °C until used for further experiments. The IASF fluid was collected from the synovial cavity using a 200 μL of phosphate buffer (PBS (pH-7.4)) solution injected into the synovial cavity of an OA knee rat. The injected PBS solution was withdrawn from the synovial cavity and stored at −80 °C for future experiments. TNF-α and IL-1β levels were measured in serum and IASF fluids using a colorimetric ELISA kit (Calbiochem-Nocabiochem Co., Milan, Italy), according to the manufacturer’s instructions [53,54].

### 2.8. FLS Cells Culture

FLS cells were isolated from the synovium knee joint. The tissues were placed in a sterilized PBS solution. The tissue surrounding fat and connective tissue was digested for 50 min at 37 °C in a collagenase (Invitrogen, Thermo Fisher Scientific, Grand Isle, NY, USA) solution (1 mg/mL). Using 70 μm cell strainers, the undigested cells were isolated from the solution. Cells were then cultured in a culture flask (25 cm^2^) with Dulbecco Modified Eagle Medium (DMEM) (Gibco BRL, NY, USA) and antibiotics penicillin and streptomycin (100 μg/mL) (Sigma-Aldrich, Darmstadt, Germany) and incubated in an incubator at 37 °C with 5% CO_2_. After reaching the confluence level, the cells were detached with 0.1% trypsin (Sigma-Aldrich, Darmstadt, Germany), and passage 8–10 numbered cells were used for all experiments. The cells were pretreated with (10 ng/mL) IL-1β before being treated with CA for 24 h under the same conditions. The MTT assay (Sigma-Aldrich, Darmstadt, Germany) was employed to determine cell viability [55].

### 2.9. Measurement of Lipid Peroxidation (LPO), Nitrate (NO), GSH, GPx, SOD, and CAT Levels

The levels of oxidative stress related biomarkers were estimated using the previous protocols [56]. In all groups, all parameters were checked in the knee OA tissue samples.

### 2.10. Western Blotting Studies

To lyse the tissue cells, RIPA lysis buffer (Beyotime, China) was used. At room temperature, the lysate was centrifuged at 12,000 rpm for 15 min. According to the previous reports, protein levels were determined using a bicinchoninic acid (BCA) protein assay (Novagen, Sigma-Aldrich, Darmstadt, Germany). Protein samples were separated on a 10% SDS-PAGE gel (50 μg protein per line) before being transferred to polyvinylidene fluoride membranes (Millipore Corporation, MA, USA). Furthermore, the membranes were then incubated for 3 h at room temperature in blocking buffer (Trisbuffer saline Tween20 (TBST) + 5% skim milk). Next, the membrane was incubated with primary antibodies overnight at 4 °C before being washed twice with TBST. Finally, the secondary antibody was incubated for 3 h. The ChemiDOCTM (Bio-Rad Laboratories, Hercules, CA, USA) was used to image the blots, and ImageJ software was used to assess band intensity [57].

### 2.11. RT-PCR Studies

The FLS cells were cultured in 100 mm cell culture dishes (10 × 10^6^ cells per dish) and used in RT-PCR studies. After CA treatment, cells were washed twice with PBS (pH-7.0), and total mRNA was extracted using TRIzol Reagent. The total mRNA (1 μg) was reverse transcripted into _C_DNA using the Prime-Script RT reagent kit and the gDNA Eraser (Takara Bio, Kusatsu, Shiga, Japan), according to the manufacturer’s instructions. Furthermore, SYBER Premix EX Taq II was used on an ABI Prism 7500 Fast RT-PCR system (Applied Biosystem, Wilmington, NC, USA). The −2^∆∆^CT method (Livak and Schmittgen) was used to calculate the expression levels, with β-actin serving as the reference gene [58]. The primer pair sequences were rat specific, as shown below Table 1.

### 2.12. Mitochondrial Membrane Permeability (ΔΨm) Assay

FLS cells were cultured in 6-well plates (1 × 10^6^ cell/well) and treated for 24 h with 50 and 100 μM CA. The dye 5,5′,6,6′-tetracholoro-1,1′,3,3′-tertraethylbenzimidazolcarboc janine iodide (JC-1) was then incubated for 30 min at 37 °C in all wells (Invitrogen, MitoProbe assay kit, NY, USA). The wells were then rinsed twice with cold PBS and imaged with a fluorescence microscope. The MateXpress-6 software was used to calculate the ratio of red and green fluorescence intensity [59].

### 2.13. DCFDA Assay

After one day of incubation, FLS cells were cultured in 6-well plates (1 × 10^6^ cells per well). The wells were treated with CA and incubated under the same conditions for another day. The DCFDA assay was used to estimate ROS production. After twice washing with PBS (pH-7.0) in the dark, DCAFDA (Sigma-Aldrich, Taiwan; Merck and Co., Neihu, Taipei, Taiwan) (10 μM) was added to each well. The fluorescence was then detected using the MateXpress-6 software [60].

### 2.14. Statistical Analysis

The mean standard deviation (mean ± S.D.) of the mean number of observations was used to express all data values in the figures and tables. Figures in the histology experiments represented all joint tissue sections from all groups, respectively. All data sets were subjected to a one-way or two-way ANOVA analysis, followed by a t-test for multiple comparisons. A *p*-value of less than 0.05 was regarded as significant. There were significant differences between the groups at * *p* < 0.05, ** *p* < 0.01, and *** *p* < 0.001.

## 3. Results

### 3.1. CA’s Impact on Weight Bearing and Knee Width in ACLT + MMx-Induced OA

**Figure 3:**
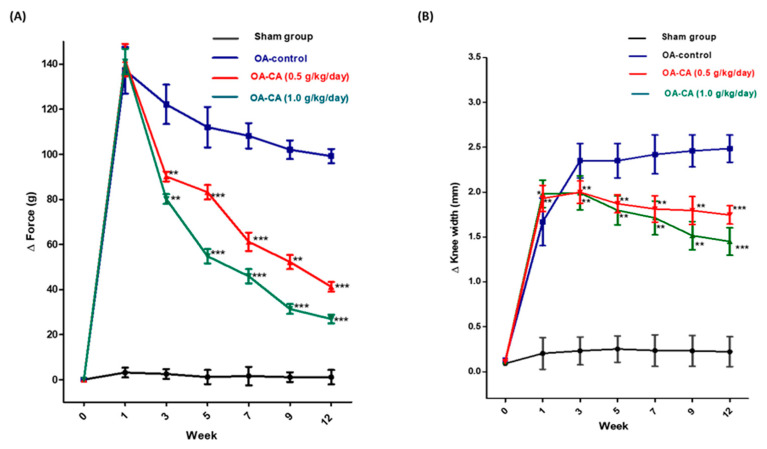
The effects of CA on (**A**) weight bearing and (**B**) knee width in ACLT + MMx induced OA knee. Measured the weight bearing and knee width of the sham, OA-control, OA-CA (0.5 g/kg/day), and OA-CA (1.0 g/kg/day) groups for 12 weeks. Data from all experiments are expressed as the mean ± S.D. * *p* < 0.05, ** *p* < 0.01, and *** *p* < 0.001.

The weight-bearing test was used to assess the pain in the knee joint. Figure 3A shows that the weight distribution difference (∆force g) between the two legs before the ACLT + MMx surgery was close to zero in all groups. The OA-control group rats had significantly higher weight distribution (∆force g) after ACLT + MMx surgery than the sham group rats. In the OA-control group, pain sensitivity increased significantly after ACLT + MMx surgery, according to these findings. When compared to the OA-control group animals, the weight distribution (∆force g) was significantly reduced after 12 weeks of daily CA administration (*p* < 0.001).

The widths of knee joints were presented in Figure 3B. After the ACLT + MMx surgery, there was a significant difference in knee width in OA-control group animals compared to the sham group animals. CA treatment significantly reduced knee swelling when compared to the OA-control group rats (*p* < 0.001). The results supported the idea that daily CA supplement reduces the swelling effect in ACLT + MMx induced OA knees.

### 3.2. CA’s Effect on Body Weight and Anti-Nociception in ACLT + MMx-Induced OA Rats

**Figure 4:**
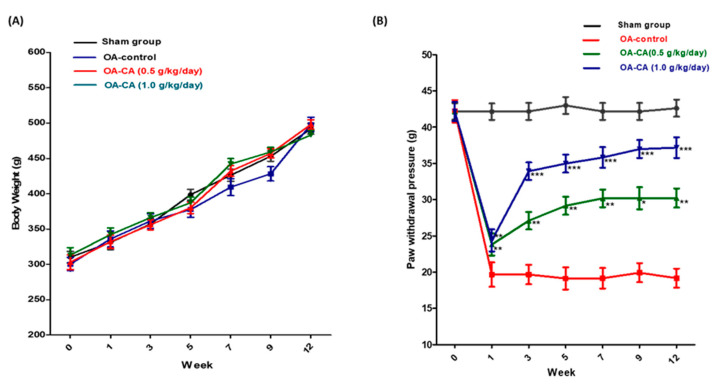
The effect of CA on (**A**) body weight changes, and (**B**) paw-withdrawal pressure in the sham group, OA-control group, and CA (1.0 and 0.5 g/kg/day) treatment groups. Data expressed as mean ± S.D. * *p* < 0.05, ** *p* < 0.01, and *** *p* < 0.001.

Throughout the experiment, no differences in body weight were observed among all the group’s animals, as shown in Figure 4A. The paw-withdrawal pressure (g) measurements of the ACLT + MMx surgery group animals were lower than those of the sham group animals, as shown in Figure 4B. after 12 weeks. CA treatment significantly increased the paw-withdrawal pressure compared to OA control group animals (*p* < 0.001). The CA could potentially alleviate mechanical allodynia pain in the animals, according to the findings.

### 3.3. The Effect of CA on Knee Histopathology Changes in ACLT + MMx-Induced OA in Rats

**Figure 5:**
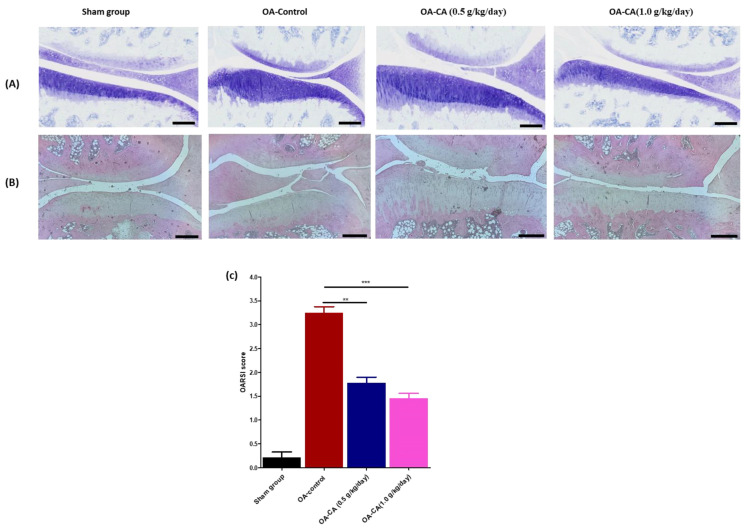
The effects of CA on histological changes and photographical representation of (**A**) toluidine blue, (**B**) H and E assay, and (**C**) calculations of OARSI scores of the sham group, OA-control, and OA-CA treated groups, scale bar = 200 μM. Experiment data expressed as mean ± S.D. ** *p* < 0.01, and *** *p* < 0.001.

Toluidine blue staining was used to detect fibrous tissue filling the cartilage defect and to assess the degree of subchondral bone loss, as shown in Figure 5A. H and E staining was used to detect cartilage and synovial membrane damage in joint tissues, as shown in Figure 5B. The severity of articular cartilage damage and further consequences of synovial membrane inflammation and damage was assessed using histopathology scores. The cartilage damage was assessed after 12 weeks of CA treatment, as shown in Figure 5A,B. The OARSI score was calculated from the H and E staining studies, as shown in Figure 5C. Toluidine blue and H and E staining studies in the OA-control group rats revealed significant damage to matrix bone loss, collapsed cartilage surface, and hyperplasia of chondrocyte cells when compared to the sham group rats.

When compared to OA-control rats, CA-treated group animals had significantly reduced cartilage matrix loss and degradation of cartilage compared. The cartilage degradation and matrix loss scores in the OA-control group were higher than in the sham group rats, according to H and E staining studies (as shown in Table 2). Furthermore, when compared with the OA-control group, the CA group rats had significantly lower total cartilage-degradation scores (*p* < 0.01). Moreover, the OA-control rats had a higher zonal depth ratio of lesions, cartilage matrix loss, and cartilage-degradation score when compared to sham group rats. In comparison to the OA control group, the CA-treated group had a significantly lower zonal depth ratio of lesions (*p* < 0.001), cartilage matrix loss (*p* < 0.05), and cartilage-degradation score (*p* < 0.01). Overall, CA supplementation significantly improves cartilage and synovial membrane protection.

### 3.4. The Effect of CA on Antioxidant Parameters on ACLT + MMx-Induced OA Knee Rats

**Figure 6:**
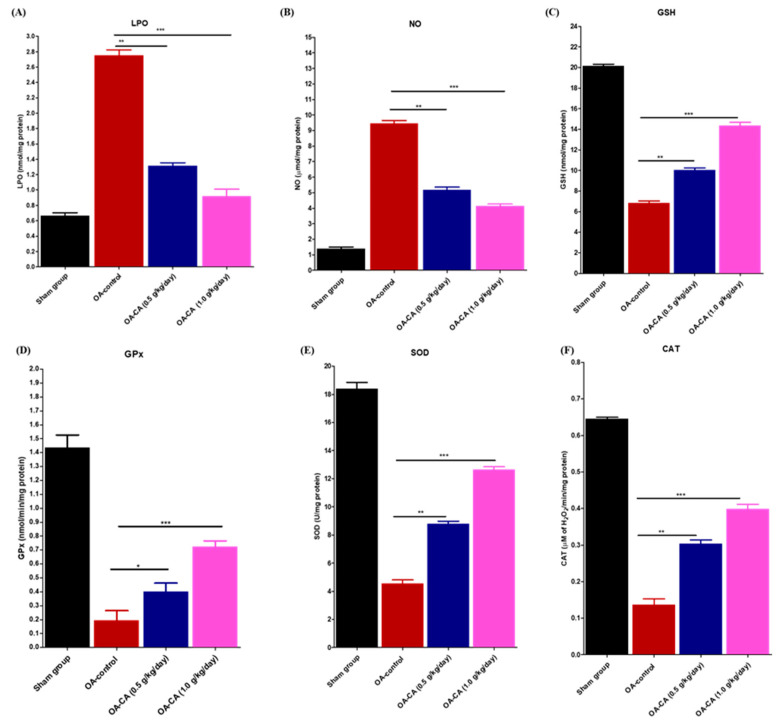
The antioxidant effect of CA was tested in the sham, OA-control, and OA-CA treated groups. The antioxidant biomarkers were measured as (**A**) LPO, (**B**) NO, (**C**) GSH, (**D**) GPx, (**E**) SOD, and (**F**) CAT and are represented in this figure. Data parameters were expressed as LPO (nmol/mg protein), NO (μmol/mg protein), GSH (nmol/mg protein), GPx (nmol/min/mg protein), SOD (U/mg protein), and CAT (μM of H_2_O_2_/min/milligram protein). Data expressed as means ± S.D. * *p* < 0.05, ** *p* < 0.01, and *** *p* < 0.001.

Figure 6A shows that there are significant differences between groups in malondialdehyde (MDA) levels in the OA-control group compared to sham group animals. MDA levels in the CA-treated group animals were significantly lower than in the OA-control group animals (*p* < 0.001). The OA-control group had significantly higher nitrate (NO) levels than the sham group animals (Figure 6B). CA treatment significantly reduced the NO levels compared to OA-control group animals (*p* < 0.001).

Furthermore, tissue GSH levels (Figure 6C) were significantly lower in OA-control animals compared to sham group animals. When compared to the OA-control group animals, CA treatment significantly enhanced the GSH levels (*p* < 0.001). Next, GPx levels (Figure 6D) were significantly lowered in comparison to sham group animals. GPx levels were higher after CA treatment compared to OA-control group animals (*p* < 0.001).

### 3.5. The Effect of CA on Serum and IASF Fluid and IL-1β and TNF-α Levels in ACLT + MMx-Induced OA Knee Rats

**Figure 7:**
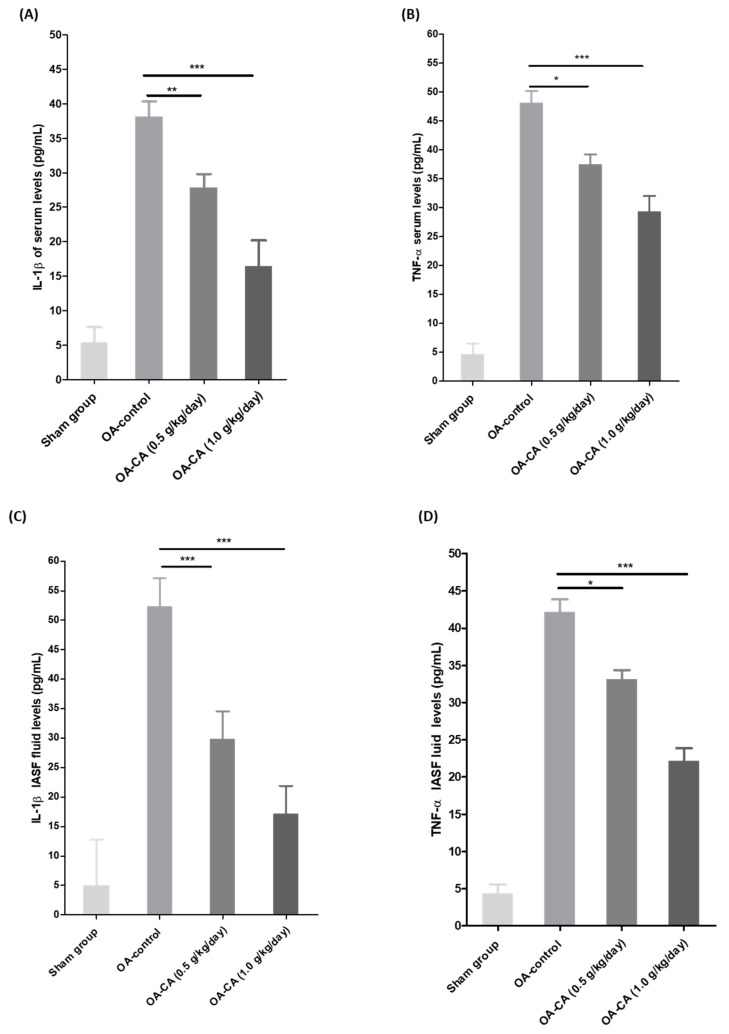
The effect of CA on the serum and IASF fluid inflammatory cytokines in sham group, OA-control, and CA-treated group animals. The serum and IASF levels of (**A**,**C**) IL-1β and (**B**,**D**) TNF-α were checked using ELISA kits. Data expressed as mean ±S.D. * *p* < 0.05, ** *p* < 0.01, and *** *p* < 0.001.

Figure 7A–D show the levels of inflammatory cytokines (IL-1β and TNF-α) in the serum and joint IASF fluids of all groups of animals. IL-1β levels in IASF were significantly higher in OA-control group animals than in sham group animals. When compared to the OA-control group animals, CA treatment significantly reduced IL-1β levels in IASF fluids (Figure 7C) (*p* < 0.001). Furthermore, TNF-α levels in IASF fluid were significantly higher in the OA-control group compared to the sham group animals. CA treatment significantly reduced IASF fluid when compared to the OA control animals (Figure 7D) (*p* < 0.001). Following that, serum IL-1β and TNF-α levels in the OA-control group animals were significantly higher than in the sham group animals (Figure 7A,B). CA treatment reduced serum IL-1β and TNF-α levels significantly (*p* < 0.001).

### 3.6. The Effect of CA on the Nrf2/HO-1 Signaling Pathway in ACLT + MMx-Induced OA Knee Rats, as Well as MMP-3 and MMP-13 mRNA Expression in FLS Cells Stimulated by IL-1β

**Figure 8:**
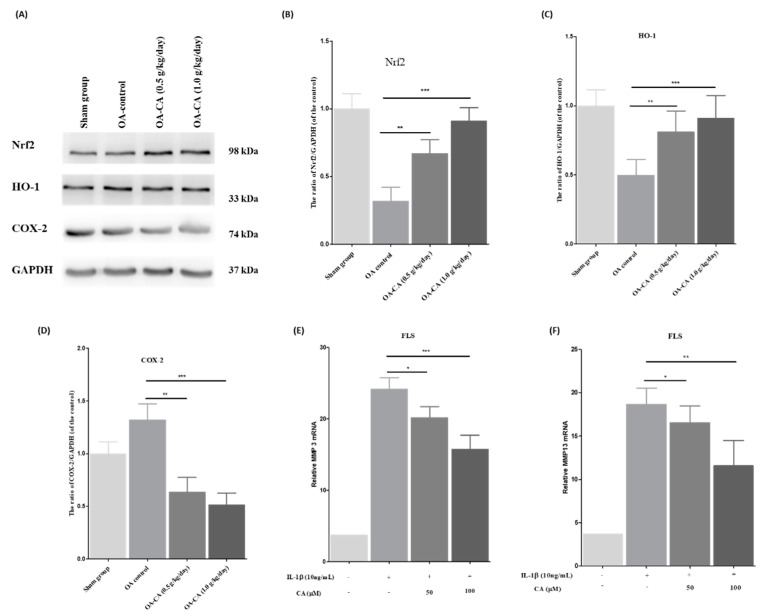
The effect of CA was checked using (**A**) Western blotting results and quantitative measurements of (**B**) Nrf2, (**C**) HO-1, and (**D**) COX-2 protein expression in sham group, OA-control, and CA-treated group animals. The data from the three individual experiments were attained; GAPDH was used as the internal standard. RT-PCR was employed for the (**E**) MMP-3 and (**F**) MMP-13 mRNA expression in the FLS cells, stimulated ROS levels with IL-1β and then treatment with CA (50 and 100 μM) for 24 h, and β-actin was used as an internal standard. Data expressed as mean ± S.D. * *p* < 0.05, ** *p* < 0.01, and *** *p* < 0.001.

The activation of the Nrf2 signaling pathway, and its consequences, were investigated through Western blot analysis of knee-tissue samples. As shown in Figure 8A, the Nrf2, and HO-1 protein levels were lower, and COX-2 protein levels were higher in the OA-control group animals compared to the sham group animals. CA treatment activates the Nrf2/HO-1 signaling pathway (*p* < 0.001), while decreasing COX-2 protein expression levels (*p* < 0.001). The quantification studies are shown in Figure 8B–D.

Furthermore, RT-PCR studies were performed to compare the levels of MMP-3 and MMP-13 mRNA expression before and after CA treatment. CA (50 and 100 μM) treatment reduced the MMP-3 and MMP-13 mRNA levels compared to IL-1β stimulated ROS FLS cells and untreated control FLS cells, as shown in Figure 8E,F.

All these findings show that CA activation of the Nrf2/HO-1 signaling pathway and the COX-2 protein level expression decreased. The Nrf2/HO-1 signaling pathway activation increased the activation of the antioxidant enzyme system, preventing ROS levels from protecting against knee OA pain. Furthermore, lowering the levels of MMP-3 and MMP-13 mRNA expression in IL-1β stimulated ROS to have FLS cells reduced knee OA pain and inflammation.

### 3.7. The Effect of CA on Cell Viability and IL-1β Stimulated ROS Production in FLS Cells

**Figure 9:**
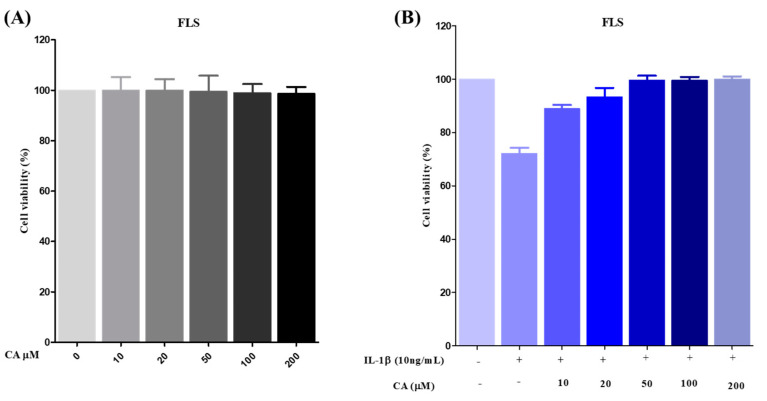
The cytotoxicity effect of CA was verified in FLS cells by (**A**) MTT assay with various concentrations of CA (0, 10, 20, 50, 100, and 200 µM), and (**B**) antioxidant capacity was checked after ROS induction in FLS by IL-1β and treatment with CA, 10 to 200 μM. All experiments were conducted in triplicates and the data were expressed as mean ± S.D.

The MMT assay was used to test the cytotoxicity of CA (0, 10, 20, 50, 100, and 200 µM) in FLS cells, as shown in Figure 9A. CA did not show any cytotoxicity after 24 h of treatment at a higher concentration of 200 µM. For further experiments, we chose 50 and 100 µM concentrations.

FLS were pretreated with IL-1β (10 ng/mL) to stimulate ROS-mediated inflammation, and then cells were treated with 10, 20, 50, 100, and 200 µM concentrations of CA for 24 h. Cell viability was remarkably reduced with CA at 50, 100, and 200 µM. These results support CA activation of the antioxidant system and a reduction in pro-inflammatory cytokines to improve cell integrity. CA treatment significantly increased the cell viability of IL-1β stimulated ROS in FLS cells (Figure 9B). These results support CA’s role as a potent antioxidant.

### 3.8. CA’s Effect on the Mitochondrial Membrane Permeability (ΔΨm) in FLS Cells Stimulated ROS with IL-1β

**Figure 10:**
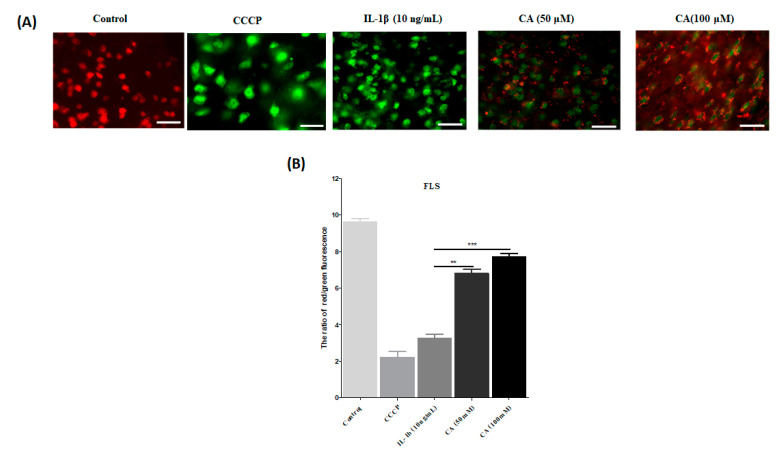
The antioxidant capacity of CA was checked in FLS by the JC-1 staining assay. (**A**) A fluorescence microscope was used to examine MMP (ΔΨm) in FLS cells exposed to CCCP (positive control), IL-1β (10 ng/mL), CA (50 and 100 μM) for 24 h and stained with JC-1 reagent to check the fluorescence, scale bar = 100 μM. (**B**) Quantitative analysis of the red to green fluorescence intensity ratio, with data expressed as mean ± S.D., ** *p* < 0.01, and *** *p* < 0.001.

That after JC-1 dye incubation, healthy mitochondria emitted red fluorescence while unhealthy mitochondria emitted green fluorescence, as shown in Figure 10A. FLS cells in the control group fluoresced red, while FLS cells in IL-1β stimulated ROS fluoresced green. This demonstrated that IL-1β successfully stimulated ROS levels and contributed to subsequent consequences, such as reducing the mitochondrial membrane permeability (ΔΨm) and antioxidant enzymes. The treatment with CA (50 and 100 μM) changed the green fluorescence to red fluorescence. It means that CA successfully enhanced the mitochondrial membrane permeability (ΔΨm). As shown in Figure 10B, the determination and quantification of the red-to-green fluorescence ratio. CA treated cells exhibit decreased green fluorescence and increased red fluorescence, indicating that CA improves mitochondrial membrane permeability (ΔΨm) (*p* < 0.001).

### 3.9. The Effect of CA on ROS Generation Was Inhibition of IL-1β Stimulated ROS Production in FLS Cells

**Figure 11:**
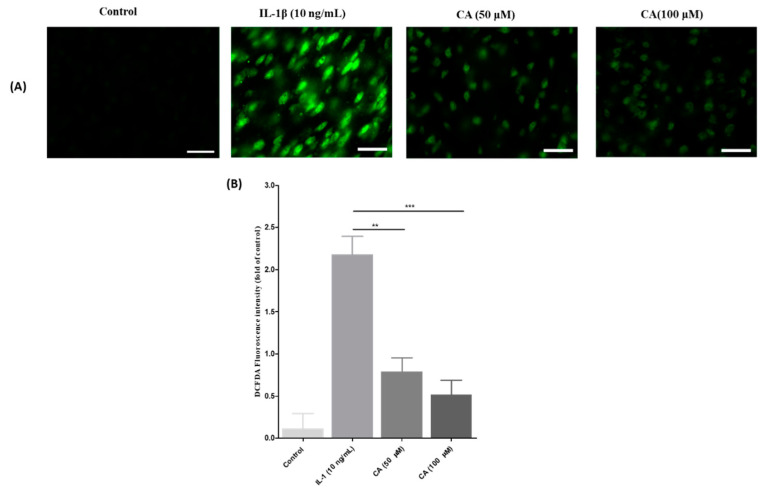
The ROS inhibition capacity of CA was checked using DCFDA assay. (**A**) Microscopic analysis of ROS generation in the FLS cells treated with IL-1β (10 ng/mL), and CA (50 and 100 μM), scale bar = 100 μM. (**B**) Quantitative assessment of DCFDA fluorescence in FLS cells. Data expressed as mean ± S.D., ** *p* < 0.01, and *** *p* < 0.001.

ROS levels were measured in this study using a DCFDA assay. ROS were stimulated in FLS cells with IL-1β (10 ng/mL) and ROS levels were significantly reduced after treatment with CA (50 and 100 μM). Figure 11A shows a decrease in green fluorescence with CA (50 and 100 μM) treatment compared to IL-1β (10 ng/mL) ROS stimulated FLS cells. Furthermore, fluorescence quantitative studies (Figure 11B) revealed a significant decrease in green fluorescence after CA treatment (50 and 100 μM) compared with IL-1β (10 ng/mL) stimulated ROS had FLS cells (*p* < 0.001).

## 4. Discussions

In vivo studies revealed that oral CA supplementation (1.0 and 0.5 g/kg/day) reduced the ACLT + MMx-induced knee OA pain while also decreasing cartilage surface erosion, synovial inflammation, and subchondral bone matrix loss. CA may have inhibited OA-induced pain and inflammation by activating the Nrf2/HO-1 signaling pathway, increasing antioxidant enzyme levels, and inhibiting the release of pro-inflammatory cytokines like IL-1β and TNF-α. CA attenuates IL-1β induced inflammation while augmenting ROS levels in FLS cells. CA has strong antioxidant potential, which helps to eradicate ROS levels. CA inhibits the inflammatory response in the ROS stimulated FLS cells by down-regulating MMP-3 and MMP-13 mRNA expression. CA was found to show great antioxidant potential in suppressing IL-1β induced ROS, as studied through the JC-1 assay and DCFDA studies, eradicating ROS levels and improving mitochondrial membrane potential.

CA is used as an antioxidant, neuroprotective, hepatoprotective, and antiaging agent [61]. CA is also known to have anti-inflammatory effects in autoimmune system inflammatory diseases, such as OA and rheumatoid arthritis [62]. Juno and colleagues investigated the protective effects of CA in streptozocin-induced diabetic neuropathic pain mice. CA treatment for nine weeks reduced nociceptive pain [63]. Another study found that the CA derivative ADM09 successfully reduced oxaliplatin-induced neuropathic pain in rats. ADM09 reduced nociceptive pain in rats via regulating the calcium connection and blocking the transient receptor potential ankyrin (TRPA1) channels [64]. In this study, the OA-control knee had a higher weight-bearing difference force (∆), and CA treatment reduced the weight-bearing difference force (∆) compared to OA-control group animals. This suggests that CA was effective in reducing knee OA pain. The weight-bearing results are consistent with the previous study [50,65], which used natural compounds to reduce knee OA-induced pain.

According to recent findings, CA inhibits amyloid beta (Aβ) induced inflammation and oxidative stress in microglial BV-2 cells via regulating the type 1 transforming growth factor (TGF-β1) receptors [66]. Ponist and colleagues investigated the CA effect of carrageenan and hydrogen peroxide-induced arthritis in rats and chondrocytes. CA successfully reduced the paw volume and hind paw swelling in rheumatoid arthritis rats in their studies [67]. The knee joint swelling measurement showed a significant difference after CA treatment, indicating that CA may inhibit knee swelling by activating the antioxidant enzyme system. CA was used as an antioxidant agent in previous OA studies. These results are consistent with previous studies [68,69]. We observed a steady weight gain in all groups over time in this study, indicating that our ACLT + MMx and CA supplements had no effect on the metabolic profile of animals. This preclinical surgery model is appropriate for knee OA research. The findings support previous reports of an ACLT + MMx-induced knee OA model in rodents [68,70].

CA conjugated hyaluronic acid compounds have been shown in recent reports to have a better protective effect in the collagen-induced arthritis rat model. Mechanical allodynia pain was measured using the paw-withdrawal method in their studies. When compared to control group animals, treatment with a CA conjugated hyaluronic acid compound significantly reduced allodynia pain [71]. Paola and colleagues investigated the protective effect of CA conjugated hyaluronic acid compound on monosodium iodoacetate (MIA)-induced OA in rats. CA conjugated with hyaluronic acid demonstrated good cartilage protection by lowering serum inflammatory cytokines and mechanical allodynia pain [72]. Chronic OA pain causes significant physiological distress and impairs physical and social functioning in patients [73]. Peripheral and chronic pain mechanisms are known to play a role in the pathophysiology of knee OA [74]. Inflammatory cytokines augmented pain sensitivity and generated action potentials in response to nociceptive neurons [75]. Paw-withdrawal pressure improved after CA treatment in this study, indicating decreased mechanical allodynia and pain nociception in ACLT + MMx induced knee OA animals.

CA has recently been investigated for its protective role, with interactions between human OA chondrocytes and 4-hydroxylnoneal (HNE) modified type II collagen affecting cell functions and phenotypes [76]. K. C. Baker and co-workers examined the metabolic serum profile after ACLT surgery, focusing on inflammatory and immune dysregulation related proteins in OA rats [77]. Chondrocytes and synoviocyte hyperplasia, as well as articular cartilage degradation, all contribute significantly to the development and progression of knee OA [78]. ACLT + MMx induced hyperplasia in chondrocytes and articular cartilage degradation in knee OA control group animals in this present study. CA treatment significantly reduced the hyperplasia and chondrocyte degradation seen in pathological staining of knee tissue.

The OARSI histological system was first described by Pritzker and colleagues [79]. Some authors have found strong links between the mankind score and the OARSI histological grades. In comparison to the mankind score, the OARSI histological grade studies focused on structural parameters [80]. The OARSI system can tell the difference between early and moderate OA, as well as subchondral bone changes in the early stage of OA. These changes track the progression of OA [81]. In this study, we found that CA treatment significantly improved cartilage protection, and reduction of cartilage matrix loss diminished the cartilage degradation score and reduced the zonal bone loss and synovial cell protection. These findings are consistent with previous research reports [82].

Zhang and co-workers investigated the protective effect of CA on salsolinol induced rat brain and rat brain epithelial cells. CA has been shown to protect the cellular structure of brain tissues in biochemical and histopathological studies [83]. They investigated the CA antioxidant capacity via inhibiting the low-density lipoprotein oxidation catalyzed by either copper or peroxyl radicals [84]. A. Yay and colleagues investigated the antioxidant effect of CA on renal oxidative stress in diabetic rats induced with streptozocin [85]. Cellular ROS is generally produced at a low level in cells and is vital for maintaining cellular homeostasis and function [86,87]. However, an imbalance in ROS levels promotes the expression of pro-inflammatory cytokines and chemokines. Excess ROS levels oxidize cellular macromolecules, such as proteins, lipids, and DNA, altering their functions [88,89,90,91]. Mitochondria, peroxisomes, and other membranous structures containing NADPH oxidase (NOXs), xanthine oxidases (XO), and nitric oxide synthase (NOS) enzymes are important sites of ROS generation [92,93]. Excess ROS production and induction of oxidative stress in chondrocytes are important tools in the pathogenesis of knee OA [94]. In humans with knee OA, augmented ROS levels caused cartilage destruction, chondrocyte hyperplasia, and synovial membrane inflammation [95]. In another study, treating primary human OA chondrocytes with IL-1β enhanced the production of cellular and mitochondrial ROS, which promotes chondrocyte apoptosis [96]. MDA and NO levels were enhanced in this study, while endogenous antioxidants, such as SOD, CAT, GSH, and GPx, levels were reduced in the joint knee tissue of OA-control group animals. CA treatment significantly reversed all the parameters via restoring the endogenous antioxidant enzymes, demonstrating that CA acts as an antioxidant agent to eradicate ROS levels.

Ohsawa and co-workers investigated the antinociceptive effect of CA in mice with inflammation-induced nociceptive effects [97]. The antioxidant capacity of CA in phorbol 12-myristate 13-acetate (PMA) caused oxidative stress and inflammation in murine macrophages in another study [42]. E. B. Yannai and colleagues investigated the antioxidant capacity of CA and N-acetyl CA compounds in microglial oxidative stress and inflammation-induced in brain cells caused by lipopolysaccharide (LPS) [98]. In the fight against infections, inflammation is a necessary cellular response [5]. Chronic and uncontrolled inflammation is linked to the pathophysiology of many human diseases, including autoimmune diseases, neurological disorders, diabetes, and bone-related diseases [99]. In the end stage of knee OA, some studies have found an increase in proinflammatory cytokines in synovial fluid [100]. OA animal models also demonstrated excessive inflammation in OA joints, supporting the case for anti-inflammatory research on knee OA treatment [101]. Recent research indicates that chondrocytes express several pro-inflammatory cytokine and chemokine receptors [102]. Accordingly, in knee OA, chondrocytes are both the source and targets of proinflammatory cytokines such as IL-1β, TNF-α and IL-6, etc. [103]. Cytokines are abundant in OA joints and are actively produced by chondrocytes and synoviocytes. Macrophages and osteoblasts play critical roles in articular cartilage degeneration, which has become a primary target for the development of new therapeutic agents [104]. In this study, we discovered high levels of IL-1β and TNF-α in serum and IASF fluids from OA control group animals. CA treatment significantly reduced the levels of IL-1β and TNF-αin serum and IASF fluids. These findings suggest that CA is a potent anti-inflammatory agent.

Wang and co-workers investigated the protective effect of CA in mouse podocytes (MPC5 cells) against high glucose-induced apoptosis via phosphatidylinositol-3-kinase (PI3K)/AKT and the Nrf2 signaling pathways [105]. CA has been shown in recent studies to successfully suppress the inflammatory response in the lipopolysaccharide-induced murine macrophage cell lines through activating the Nrf2 signaling pathway [106]. X. Q. Wang and colleagues investigated the CA’s ability to protect intestinal stem cells from deoxynivalenol-induced oxidative stress via activating the Nrf2 signaling pathway [107]. The Nrf2 signaling pathway is involved in the regulation of oxidative stress, inflammation, immunity, bone metabolism, and cartilage [108]. In rodent pain models, activating the Nrf2 signaling pathway reduced inflammatory and neuropathic pain [109]. In a recent study, a sulforaphane-activated Nrf2 signaling pathway was found to reduce allodynia and hyperalgesia while augmenting morphine’s antinociception [110]. Furthermore, a recent study found that activating Nrf2 reduced nociceptive hypersensitivity by reducing mitochondrial dysfunction and neuro-inflammation in a dimethyl fumarate-induced peripheral nerve injury model [111]. These findings suggest that the Nrf2 signaling pathway may be a promising therapeutic approach for chronic pain associated with knee OA treatment [112,113,114]. In this study, we found that in OA-control rats, Nrf2 and HO-1 proteins were down-regulated, but COX-2 was up-regulated. When compared to the OA-control group rats, CA significantly activates the Nrf2 and HO-1 proteins while down-regulating the COX-2 protein levels. These findings indicate that CA successfully activated the antioxidant enzyme cascade to protect the knee tissue cells from the damaging effects of ROS. As a result, the current study assessed CA protective activity via Nrf2 signaling pathway activation. Antioxidant enzymes restored and alleviated knee OA pain.

Bae and colleagues investigated whether CA influences MMP activity in rats with cerebral ischemic injury. The gelatin zymology assay was used to measure MMP levels in the brain [115]. In another study, researchers investigated the effects of CA and gallic acid synesthetic peptides on MMP-2 and MMP-9 in human fibrosarcoma HT1080 cells [116]. CA has been shown in recent studies to inhibit metastasis in SK-Hep-1 cells via inhibiting MMP proteins and an antimetastatic gene (nm23-H1) [117]. Targeting FLS-mediated synovial inflammation may be a new therapeutic avenue for OA treatment, according to growing evidence [118]. MMP-3 and MMP-13 proteins are important biomarkers in the extracellular matrix of cartilage and play an important role in the development and progression of OA [119]. FLS cells contain MMP’s as well as various surface adhesion molecules [120]. Thus, after stimulating ROS levels with IL-1β, this study examined the expression of MMP’s levels in FLS cells. Interestingly, IL-1β stimulated FLS cells augmented the levels of MMP-3 and MMP-13 mRNA. MMP-3 and MMP-13 mRNA levels in FLS were reduced after CA treatment. This result supports the hypothesis that CA specifically inhibits the inflammatory response in ROS stimulated FLS cells via inhibiting the synthesis of MMP-3 and MMP-13 proteins.

Gaunitz and colleagues investigated the CA inhibitory effect in cancer patients with glioblastoma [121]. In recent studies, the inhibitory effect of CA was induced by the 6-hyroxydopamine-induced endoplasmic stress in SH-SY5Y cells [122]. In another study, they investigated the CA’s ability to protect against amyloid beta-42 induced neurotoxicity in rat PC12 cells. CA improved cell viability in PC12 cells in a concentration-dependent manner, according to their findings [123]. In recent reports, they investigated the CA antiglycation and antioxidant effect in glucose degradation against human peritoneal mesothelial cells [124]. FLS cells were extracted from rat joints, releasing large amounts of ROS in response to IL-1β biochemical stimuli, which would increase the production of ROS [125]. This study investigated the CA protective effect in the FLS cells as well as IL-1β induced ROS have FLS cells. CA (10 to 200 μM) treatment at high concentrations did not cause toxicity in FLS cells. Therefore, we chose 50 and 100 μM for further FLS cell-based research. FLS cells recovered from IL-1β induced ROS cell number reduction in a concentration-dependent manner. This result supports the argument that CA successfully reduced ROS levels while also protecting FLS cells from the harsh environment of ROS via antioxidant action.

Kim et al. investigated CA’s therapeutic effect against human cervical carcinoma cells and Mandin-Darby kidney cells (MDCK). CA reduced the apoptosis, DNA damage, and ROS concentration in cancer cells, according to cell-based studies. CA restores mitochondrial membrane potential and reduces ROS production while escalating cell viability [126]. Recent studies investigated the nephroprotective effect of CA on ifosfamide-induced renal injury and electrolyte imbalance via mitochondrial membrane function regulation and oxidative stress inhibition [127]. Omati et al. investigated CA’s neuroprotective effects in manganese-induced neurotoxicity in mice by altering the mitochondrial membrane potential and inhibiting oxidative stress [128]. The mitochondrial membrane potential in IL-1β induced enhanced ROS in FLS cells was discovered in this study. ROS-stimulated FLS cells had lower mitochondrial membrane potential (ΔΨm), but CA treatment significantly improved the mitochondrial membrane potential (ΔΨm), indicating that CA may have reduced the ROS induced oxidative stress in FLS cells.

Mahmood et al. investigated the antioxidant effect of CA in human blood cells against copper-induced cytotoxicity and oxidative stress [129]. CA may protect brain microvascular endothelial cells from rotenone-induced oxidative stress via histamine receptors, according to Chen and co-workers [130]. CA has been shown in recent studies to protect impaired erythrocytes from oxidative stress caused by hydrogen peroxide [131]. In this study, CA eradicated ROS against IL-1β induced oxidative stress in FLS cells in a dose-dependent manner.

## 5. Conclusions

We demonstrated the CA inhibition effect against knee OA in this study using in vivo and in vitro studies. CA reduced post-surgery pain in rats, according to preliminary pain-related behavioral studies. Furthermore, CA inhibited the knee’s OA-related pro-inflammatory cytokines. CA reduced cartilage damage, enhancing synovium membrane protection. Next, we identified that CA could activate the Nrf2/HO-1 signaling pathway, decrease COX-2 expression, and restore antioxidant enzymes. CA reduced the expression of MMP-3 and MMP-13 mRNA in IL-1β stimulated ROS in FLS cells. Furthermore, CA successfully eradicated the IL-1β induced augmented ROS and enhanced the mitochondrial membrane potential. On this basis, CA protects the synovium membrane and alleviates pain associated with knee OA. 

## Figures and Tables

**Table 1:** The primer pair sequences of β-actin, MMP-3, and MMP-13 genes.

Gene		Primer Sequence (5′-3′)
MMP-3:	F	5′-CATAATACACAGCTGACCTGTATAA-3′
	R	5′-ATTTAAGAAATCATAGATAACAGTTACTTA-3′
MMP-13:	F	5′-TGATGATGAAACCTGGACAAGCA-3
	R	5′-GAACGTCATCTCTGGGAGCA-3′
β-actin:	F	5′-GGAGATTACCTGCCCTGGCTCCTA-3′
	R	5′GACTCATCTACTCCTGCTTGCTG-3′

**Table 2:** Detection of histopathology changes in the sham group, and OA-control group, OA-CA treated group animals. Histopathology changes were examined from the H and E staining samples from all group animals. Data expressed as mean ± S.D. * *p* < 0.05, ** *p* < 0.01, and *** *p* < 0.001.

Histopathological Changes in the Knees	Sham Group (*n* = 5)	OA-Control (*n* = 5)	OA-CA (0.5 g/kg/day) (*n* = 5)	OA-CA (1.0 g/kg/day) (*n* = 5)
Cartilage matrix loss (mm)	0 **	4.0 ± 0.6	3.1 ± 0.7 *	2.4 ± 0.3 *
2. Cartilage degeneration score	0 ***	11.1 ± 1.6	5.91 ± 1.5 ***	3.2 ± 0.2 **
3. Total cartilage degeneration width(mm)	0 ***	2.19 ± 0.6	1.85 ± 0.2 **	1.13 ± 0.1 **
4. Significant cartilage degeneration width (mm)	0 ***	1.9 ± 0.23	0.9 ± 0.6 *	0.3 ± 0.1 ***
5. Zonal depth ratio of lesions	0 **	2.1 ± 0.4	0.9 ± 0.5 *	0.5 ± 0.4 **

## Data Availability

Data is contained within the article.
